# Breast Cancer-Related Lymphedema (BCRL): Comprehensive Characterization of Patients Seeking Microsurgical Treatment

**DOI:** 10.1245/s10434-025-18694-8

**Published:** 2025-11-18

**Authors:** Daphne van Gemert, Louise Marie Beelen, Julia Mos, Laurentine van Egdom, Gilbert-Jan van Gaalen, Agnes Jager, Linetta Koppert, Anne-Margreet van Dishoeck, Mark van der Oest, Dalibor Vasilic

**Affiliations:** 1 Department of Plastic and Reconstructive Surgery, Erasmus MC University Medical Center, Rotterdam, The Netherlands; 2 Department of Medical Oncology, Erasmus MC Cancer Institute, Rotterdam, The Netherlands; 3 Department of Surgery, Erasmus MC University Medical Center, Rotterdam, The Netherlands; 4 Department of Research and Education, Erasmus MC University Medical Center, Rotterdam, The Netherlands

**Keywords:** Breast cancer, Breast cancer-related lymphedema, Microsurgery, Oncology, Secondary lymphedema

## Abstract

**Background:**

Accurate characterization of the breast cancer-related lymphedema (BCLR) population is essential to understand its pathophysiology and develop predictive models for identifying at-risk patients and implementing tailored preventive microsurgical strategies. Key factors influencing BCRL severity and progression remain unclear. This study characterizes patients with BCRL seeking microsurgical treatment and evaluates the impact of oncological treatment intensity on lymphedema severity and progression.

**Methods:**

This cohort study was conducted at an outpatient tertiary lymphedema clinic between 2017 and 2023. BCRL severity was assessed at intake by a lymphedema-specialized plastic surgeon using International Society of Lymphology staging and indocyanine green lymphography with near-infrared fluorescence imaging (ICG-NIFI). Data were collected during scheduled medical evaluations and analyzed retrospectively. Exploratory analysis investigated associations between oncological treatment intensity and BCRL severity and progression.

**Results:**

A total of 163 consecutive female patients with BCRL were included. Lymphedema severity varied significantly, with no consistent link between severity and time since onset. A significant association was found between axillary lymph node dissection (ALND) and ICG-NIFI stages (p<0.001). However, no significant associations were found between oncological treatment intensity—surgery, radiotherapy, systemic treatment—and BCRL severity and progression. Analyses further revealed associations between lymphedema severity, body mass index, postmenopausal status, and clinical course.

**Conclusion:**

This study provides a comprehensive profile of patients with BCRL seeking microsurgical treatment, revealing variable lymphedema progression patterns. Oncological treatment intensity did not appear to influence BCRL severity or progression, suggesting that these may depend more on biological predisposition. These findings enhance BCRL understanding and highlight the importance of precise patient characterization, laying the foundation for targeted, individually tailored preventive microsurgical interventions.

**Supplementary Information:**

The online version contains supplementary material available at 10.1245/s10434-025-18694-8.

Breast cancer is the most common cancer among women worldwide, recording 2.3 million new cases in 2020 and projections indicating a rise to over 3 million annually by 2040.^1^ Despite advancements in cancer care and personalized medicine, long-term complications from the disease and its treatments persist. One of these complications is breast cancer-related lymphedema (BCRL), which affects 20–41% of patients after treatment.^2^

This chronic and progressive condition results from disruption of the lymphatic system, after which fluid accumulation, inflammation, and swelling lead to hardening of the skin, fibrosis, and deformation of the affected body part, resulting in reduced mobility and loss of function.^3–5^These structural disruptions are believed to arise after procedures such as axillary lymph node dissection (ALND), axilla-preserving surgeries such as sentinel lymph node biopsy (SLNB) and targeted axillary dissection, radiotherapy, or surgery and radiotherapy combined.^6^ Documented patient-related risk factors include high body mass index (BMI), subclinical edema, and cellulitis of the affected limb.^7^ BCRL causes symptoms such as swelling, pain, impaired arm function, and infections, in turn significantly degrading quality of life (QoL),^8,9^ often forcing patients to reduce working hours or stop working, affecting their economic stability.^9,10^

BCRL is typically managed through decongestive therapy, involving conservative techniques such as compression therapy, manual lymphatic drainage, bandaging, taping, massage, and exercise. When conservative methods prove to be insufficient, (micro)surgical interventions such as lymphaticovenous anastomosis (LVA) and vascularized lymph node transfer (VLNT) may be considered. These microsurgical techniques have demonstrated potential in alleviating symptoms and improving QoL, but complete restoration of lymphatic function has not yet been achieved and treatment remains focused on symptom management.^11,12^

Accurate characterization of the BCRL population is crucial for enhancing knowledge of its pathophysiology and epidemiology and for developing predictive models to identify at-risk patients. This understanding is crucial for implementing preventive strategies such as immediate lymphatic reconstruction in a healthcare field challenged by economic and expertise constraints, while still aiming to reduce the healthcare burden and improve patient QoL.^13^

This study aims to establish a comprehensive characterization of the BCRL population seeking microsurgical treatment and investigate associations between breast cancer treatment intensity and lymphedema progression and severity.

## Methods

A prospective database was created of patients with BCRL seeking microsurgical treatment at a university medical center’s outpatient clinic in the Netherlands. Data from 2017 to 2023 were collected during scheduled medical evaluations and extracted from electronic health records.

### Clinical Assessment

BCRL severity was assessed by a lymphedema-specialized plastic surgeon using International Society of Lymphology (ISL) staging, incorporating limb circumference measurements and clinical symptoms.^14^ Indocyanine green lymphography with near-infrared fluorescence imaging (ICG-NIFI) was performed at intake and classified per MD Anderson BCRL staging.^15^ ICG-NIFI staging (I–V) evaluates lymphatic patency and dermal backflow, with stage I indicating numerous patent lymphatics and minimal backflow, whereas higher stages reflect progressive decline in lymphatic patency and increased dermal backflow.^15^ Stage IV is characterized by dermal backflow involving the hand, and stage V represents complete absence of ICG movement past the ICG injection site.^15^ Microsurgical recommendations were based on staging, progression, and symptoms. LVA was typically indicated for ICG-NIFI stages II–III, whereas patients at stage IV were recommended VLNT. Lymphedema progression was assessed through patient self-report, tracking onset from initial breast cancer treatment to microsurgery consultation, based on changes in limb diameter, pain, heaviness, and fatigue.

### Data Collection

At intake, patients completed patient-reported outcome measure questionnaires, including the Lymphoedema functioning, disability and health questionnaire (Lymph-ICF),^16^ the EQ5D-5L,^17^ the Disabilities of the Arm, Shoulder and Hand (DASH) questionnaire,^18^ and the Michigan Hand Outcomes Questionnaire (MHQ).^19^ Reviewed data included patient characteristics, lymphedema-specific information, and oncological history. Patient characteristics covered demographics, comorbidities, intoxications, and referral information. Lymphedema characteristics included affected regions, triggers, clinical progression, prior conservative and microsurgical treatments, infections, severity according to ISL staging,^14^ and ICG-NIFI staging categorized per MD Anderson staging.^15^ Oncological history covered tumor pathology, TNM staging, DNA mutations, surgical outcomes, and details of radiation and systemic therapies. Data usage was approved under an opt-out consent model.

### Statistical Analysis

A power analysis determined a sample size of 163 to detect effect sizes over 0.35 (*α* = 0.05, *β* = 0.8). Data analysis was performed with Rstudio version 4.3.1, and missing data were addressed through multivariate imputation by chained equations using the “mice” package.^20^ Descriptive statistics were used for data summarization, presenting nominal and ordinal variables in frequencies and percentages, and continuous variables as means with standard deviation (SD) or medians with interquartile range (IQR). Exploratory analyses evaluated associations between breast cancer treatment modalities and BCRL severity and progression. Shapiro–Wilk tests were used to assess normality; non-normal distributions were described using medians and IQRs. Kruskal–Wallis and analysis of variance tests were used to analyze variable distributions, with Dunn and Tukey post hoc tests for significant findings. Associations between categorical variables were investigated using chi-squared tests. Bonferroni correction adjusted for multiple testing, setting significance thresholds at *p*<0.00119 for primary analyses and *p*<0.05 for secondary analyses.

## Results

### Patient Characteristics

Baseline characteristics are displayed in Table 1. This cohort study included 163 female patients with an average ± SD age of 59.4 ± 11.6 years and a mean ± SD BMI of 28.3 ± 5.03 kg/m^2^. Most patients (89%) were referred by specialists; 11% were referred by general practitioners.

**Table 1:** Baseline characteristics

Characteristic	Overall (N=163)
Age	60.0 (51.0–66.0)
Sex, female	163 (100)
BMI (kg/m^2^)	27.8 (24.7–31.2)
*Menopausal status*	
Premenopausal	15 (9.2)
Perimenopausal	24 (14.7)
Postmenopausal	124 (76.1)
*Dominance*	
Right	137 (84.0)
Left	23 (14.1)
Ambidextrous	3 (1.8)
*Smoking*	
No	107 (65.6)
Stopped smoking	48 (29.4)
Yes, actively	8 (4.9)
Alcohol (yes)	55 (33.7)
*Comorbidities (yes***)**	
Hypertension	44 (27)
Cardiovascular disease	18 (11)
Diabetes mellitus	14 (8.6)

Data are presented as median (interquartile range) or n (%) unless otherwise indicated

BMI, body mass index

### Breast Cancer Treatment

**Fig. 1:**
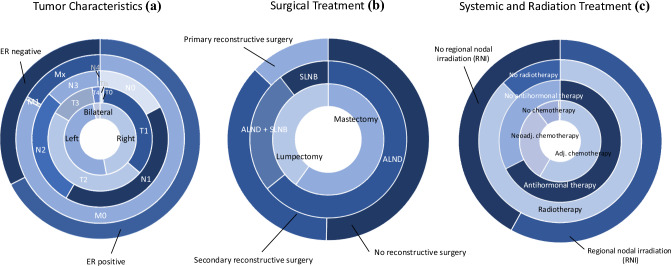
Multi-layered pie charts illustrating **a** tumor characteristics, **b** surgical treatment, and **c** systemic and radiation treatments in patient breast cancer histories. Adj, adjuvant; ALND, axillary lymph node dissection; ER, estrogen receptor; SLNB, sentinel lymph node biopsy

Oncological characteristics are summarized in Fig 1 (full data in Supplementary Table 1). The upper outer quadrant was the most common primary tumor location (65.6%), followed by the upper medial quadrant (14.7%). Nearly all patients (98.8%) underwent surgical removal of oncological tissue, with mastectomies representing 60.2% and breast-conserving surgery 39.8% of procedures. Reconstructive surgery was performed in 50.3% of mastectomies. Axillary surgery was performed in 99.4% of patients, with 55.2% undergoing ALND, 34.4% both ALND and SLNB, and 9.8% only SLNB.

In this study, 147 of 163 patients (90.2%) underwent chemotherapy; 34.7% received neoadjuvant, 64.6% adjuvant, and 0.7% both. The most common chemotherapy regimens were a combination of anthracycline and taxane-based regimens (63.2%), followed by solely anthracycline-based (23.9%) regimens and solely taxane-based regimens (3.1%). Taxane-based chemotherapy was used in 66.3% of cases, whereas platinum-based chemotherapy was used in 4.9%. Antihormonal therapy, primarily tamoxifen, was administered in 67.5% of cases. HER2-targeted therapy, specifically monoclonal antibodies, was administered to 19 patients (11.7%), all of whom received trastuzumab, with two also receiving pertuzumab.

Radiotherapy was administered to 87.7% of patients, targeting the breast or chest wall, axilla, and associated lymphatic regions. The median number of sessions was 25 (range 0–33), with a median radiation dose of 50 Gy (range 0–70), and 27.6% of patients receiving an additional radiation boost.

### Lymphedema Characteristics

Lymphedema characteristics are presented in Table 2. BCRL affected the right side in 50.3% of patients and the left side in 49.1%, with bilateral involvement in only one patient (0.6%). The years since lymphedema onset ranged from 1 to 25 years (median 7). The median time from breast cancer diagnosis to lymphedema onset was 1 year (IQR 0–2).

**Table 2:** Lymphedema characteristics

Characteristics	Overall (N=163)
Years since BCRL onset	7 (5–11)
Time between breast cancer diagnosis and lymphedema onset	1 (0–2)
*Affected side*	
Right	82 (50.3)
Left	80 (49.1)
Bilateral	1 (0.6)
*ISL – stage*	
Stage I	16 (9.8)
Stage IIa	41 (25.2)
Stage IIb	76 (46.6)
Stage III	30 (18.4)
*ICG result*	
Stage 1	17 (10.4)
Stage 2	59 (36.2)
Stage 3	50 (30.7)
Stage 4	37 (22.7)
*Affected locations *	
Hand	81 (49.7)
Lower arm	148 (90.8)
Upper arm	151 (92.6)
Shoulder	5 (3.1)
Supraclavicular region	3 (1.8)
Breast	22 (13.5)
Flank	19 (11.7)
Multiple affected locations (yes)	150 (92.0)
Pitting edema (yes)	106 (65.0)
Infections in the affected limb (yes)	55 (33.7)
*Clinical course*^*a*^	
Decrease	3 (1.8)
Stable	78 (47.9)
Gradual increase	75 (46)
Rapid increase	7 (4.3)

Data are presented as median (interquartile range) or n (%) unless otherwise indicated

BCRL, breast cancer-related lymphedema; ICG, indocyanine green lymphography; ISL, International Society of Lymphology

^a^Changes in clinical symptoms since intake, categorized as decrease, stable, gradual increase, or rapid increase

**Fig. 2:**
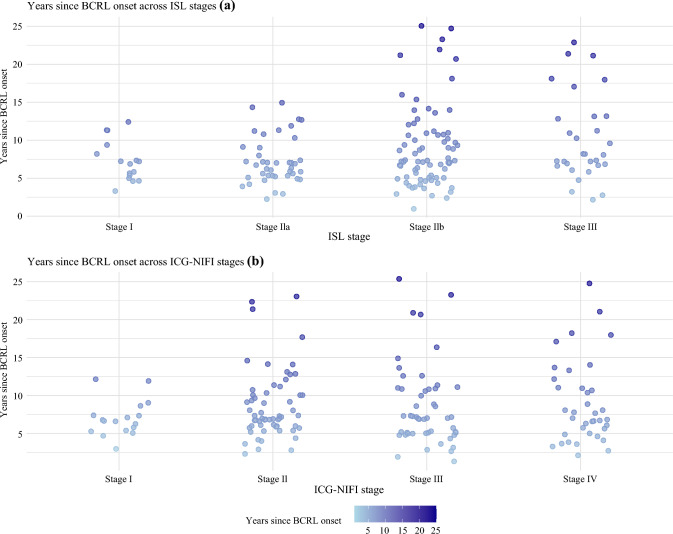
Years since breast cancer-related lymphedema (BCRL) onset in relation to BCRL severity across **a** International Society of Lymphology (ISL) and **b** indocyanine green lymphography with near-infrared fluorescence imaging (ICG-NIFI) stages

BCRL severity, assessed by ISL and ICG-NIFI staging, varied regardless of time since onset Fig 2. Patient-reported events correlating with lymphedema onset included radiotherapy (22.7%), breast surgery (13.5%), and ALND (7.4%). Among the patients attributing it to radiotherapy (*n* = 37), 32.4% had received additional radiation boosts. Of those linking it to surgery (*n*= 22), 68.2% (n = 15) underwent mastectomy and 31.8% (*n* = 7) had lumpectomy. In 37.4% (*n* = 61), the onset of lymphedema could not be correlated to a specific event.

**Fig. 3:**
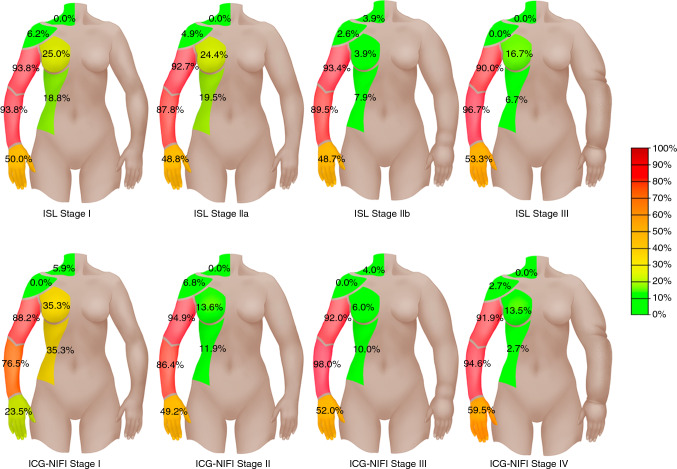
The gradient illustrates the distribution pattern of breast cancer-related lymphedema (BCRL) across body parts in relation to International Society of Lymphology (ISL) and indocyanine green lymphography with near-infrared fluorescence imaging (ICG-NIFI) stages. The left arm of each torso depicts the clinically observable changes in the affected body parts with increasing severity

Fig 3 illustrates lymphedema distribution across various body parts, organized by ISL and ICG-NIFI staging. The most commonly affected areas were the lower and upper arm, observed in 148 (90.8%) and 151 patients (92.6%), respectively. The majority of patients (92%) reported BCRL affecting multiple body parts. A pattern of increasing lymphedema involvement with severity was evident: in the lower stages, lymphedema primarily affected the lower and upper arms, with less involvement of the hand, breast, and flank areas. By ISL stage III, lymphedema prevalence in the upper and lower arm increased to 90.0% and 96.7%, respectively. Hand involvement increased from 23.5% in ICG-NIFI stage I to 59.5% in stage IV. Breast and flank involvement remained lower throughout advancing stages. The shoulder and supraclavicular region were minimally affected across all stages.

Changes in limb diameter, pain, heaviness, and tiredness were recorded as changes in clinical course or progression. Most patients reported either a stable course (47.9%) or a gradual increase (46.0%) in symptoms, and few experienced a decrease (1.8%) or a rapid increase (4.3%).

Regarding lymphedema management, 93.9% had received conservative treatments before considering microsurgery. The most common conservative treatment modalities consisted of compression sleeves and manual lymphatic drainage, with 63.8% of patients using a combination of techniques, including wrapping, pneumatic compression, hyperbaric oxygen therapy, and alternative medicine.

### Microsurgical Treatment Recommendation

Microsurgical treatment recommendations are described in Table 3. The majority of the patients (80.4%) were advised to receive microsurgical treatment. LVA was the most frequently recommended procedure, suggested for 100 patients (76.3%). Additionally, 23 patients (17.6%) were recommended free VLNT, and eight (6%) were advised to have a combination of deep inferior epigastric perforator flap with either LVA or VLNT.

**Table 3:** Microsurgical treatment recommendation

Recommendation	Overall (N=163)
Microsurgical intervention (yes)	131 (80.4)
Microsurgical treatment recommendation	
LVA	100 (76.3)
VLNT	23 (17.5)
DIEP + LVA	4 (3.1)
DIEP + VLNT	4 (3.1)

Data are presented as n (%)

DIEP, deep inferior epigastric perforator; LVA, lymphaticovenous anastomosis; VLNT, vascularized lymph node transfer

### Patient-Reported Outcome Measures at Intake

The mean ± SD Lymph-ICF score was 38.9 ± 17.3, categorized by severity: one patient (0.6%) had “no problem”, 34 patients (20.9%) had “small problem”, 80 (49.1%) “moderate problem”, and 48 patients (29.4%) had “severe problem” (Supplementary Figure 1). EQ5D-5L index scores, which are measured on a fixed scale from −0.59 to 1, ranged from −0.158 to 1 within this cohort, with a median of 0.739 (IQR 0.6–0.8). DASH scores ranged from 2.5 to 83.3 with a median score of 34.2 (IQR 18.8–45.8). MHQ total scores ranged from 8.75 to 100, with a median score of 62.4 (IQR 47.9–78.8).

### Exploratory Statistical Analysis

Primary univariate exploratory analyses Table 4 investigated associations between breast cancer treatment modalities and BCRL severity and progression. No significant associations were found between breast surgery type and BCRL severity across ISL (*p* = 0.89) and ICG-NIFI (*p* = 0.35) stages or clinical progression (*p* = 0.83). ALND was significantly associated with higher ICG-NIFI stages (*p*<0.001), with significant differences persisting between stage I and II and stages I and III after Bonferroni correction (α_corrected_ = 0.00119). SLNB showed no significant association with ISL stages (*p* = 0.06). An initially significant association between regional nodal irradiation and ISL stages (*p* = 0.01) did not remain significant after Bonferroni correction. Similarly, although the total number of lymph nodes removed was associated with ICG-NIFI stages (*p* = 0.04), this significance did not hold after applying the Bonferroni correction.

**Table 4:** Exploratory statistical analyses evaluating associations between breast cancer treatment intensity, breast cancer-related lymphedema (BCRL) severity and progression

Variable	ISL stage		ICG-NIFI stage		Clinical course^**a**^	
	χ^2^ or H	*p*	χ^2^ or H	*p*	χ^2^ or H	*p*
*Primary analyses*						
Breast surgery ^b^	1.286	0.733	2.743	0.433	0.547	0.908
Axillary lymph node dissection	6.215	0.102	17.222	**<**0.001^c^	2.507	0.474
Sentinel lymph node biopsy	5.744	0.125	8.672	0.039	4.985	0.056
Lymph nodes removed (total)	1.926	0.588	8.379	0.038	0.179	0.981
Radiotherapy	2.045	0.563	0.438	0.932	0.473	0.925
Regional nodal irradiation	11.063	0.011	0.127	0.988	0.806	0.848
Radiotherapy boost	3.005	0.808	8.104	0.231	0.727	0.994
High radiation dosage (>60 Gy)	2.267	0.519	2.656	0.448	4.025	0.259
Chemotherapy	0.464	0.927	8.137	0.202	3.134	0.371
Neoadjuvant or adjuvant	4.839	0.848	8.070	0.527	25.882	0.002
Number of chemotherapy sessions	1.918	0.590	2.718	0.437	2.183	0.535
Taxane-based chemotherapy	1.863	0.601	1.615	0.656	0.116	0.989
Breast reconstructive surgery	2.729	0.435	1.639	0.651	1.585	0.663
Monoclonal antibodies (anti-HER2)	1.980	0.577	6.433	0.092	4.191	0.242
*Secondary analyses*						
Body mass index	9.497	0.023	4.827	0.185	9.282	0.026
Postmenopausal status	16.533	**<**0.001	3.098	0.377	3.409	0.333
Clinical course	6.611	0.678	25.347	0.003	NA	NA

^a^Decline, stable, gradual increase, rapid increase in clinical symptoms

^b^Mastectomy or lumpectomy

^c^Statistically significant association after Bonferroni correction

HER2, human epidermal growth factor receptor 2; ICG-NIFI, indocyanine green lymphography with near-infrared fluorescence imaging; ISL, International Society of Lymphology

No significant associations were found between radiotherapy and ISL (*p* = 0.56) or ICG-NIFI stages (*p* = 0.93) and clinical progression (*p* = 0.93). Similarly, the application of radiation boosts, as well as radiation dosages exceeding 60 Gy, revealed no significant associations with ISL (*p* = 0.81; *p* = 0.52), ICG-NIFI stages (*p* = 0.23; *p* = 0.45), and clinical progression (*p* = 0.99; *p* = 0.26). Chemotherapy, regardless of timing or the number of sessions, demonstrated no significant association with ISL (*p* = 0.93; *p* = 0.85; *p* = 0.59) and ICG-NIFI stages (*p* = 0.20; *p* = 0.53; *p* = 0.44). Initial findings linking chemotherapy timing (adjuvant or neoadjuvant) and clinical course (*p* = 0.002) did not hold after Bonferroni correction. Taxane-based chemotherapy showed no significant effects on ISL (p = 0.60) and ICG-NIFI stages (*p* = 0.66) or clinical progression (*p* = 0.99). Breast reconstructive surgery also showed no significant effects on ISL (*p* = 0.44) and ICG-NIFI stages (*p* = 0.65) or clinical progression (*p* = 0.66). Secondary exploratory analyses Table 3 revealed significant associations between BMI and both ISL stages and clinical course (*p* = 0.023; *p* = 0.026), postmenopausal status and ISL stages (*p*<0.001), and clinical course and ICG-NIFI stages (*p* = 0.003).

### Patient-Reported Outcome Measures

MHQ total scores varied significantly across ISL stages, with stage III scoring notably lower than stage I (mean difference −18.28; 95% confidence interval [CI] −34.99 to −1.57) and stage IIa (mean difference −13.38; 95% CI −26.35 to −0.41). Analysis of variance tests showed significant variations in Lymph-ICF, DASH, and EQ5D-5L scores with clinical progression. Post-hoc analysis highlighted significant differences in Lymph-ICF scores between “stable” and “gradual increase” groups (*p* = 0.004), in EQ5D-5L scores between “stable” and both “gradual increase” (*p* = 0.02) and “rapid increase” groups (*p* = 0.045), and in DASH between “stable” and “gradual increase” groups (*p* = 0.01). No associations were found between radiation dosage, chemotherapy sessions, and patient-reported QoL.

## Discussion

This study provides a comprehensive characterization of patients with BCRL seeking microsurgical treatment at a tertiary care center. Although this cohort represents a selective, high-risk population, its characterization holds considerable clinical relevance because these are precisely the patient profiles that need to be identified earlier in the course of treatment to enable timely implementation of preventive strategies. As interest in LYMPHA and other preventive interventions continues to grow, a significant gap remains in the understanding of the patient population at risk. This study helps lessen the gap by outlining the lymphedema characteristics and treatment profiles of a population already referred for microsurgical treatment.

Compared with the general Dutch breast cancer population, patients in this cohort underwent more intensive oncological treatment.^21^ National trends over the past 25 years have shown a shift toward SLNB as the standard surgical axillary approach, with ALND rates decreasing from 70% in 2000 to 4% in 2023, and SLNB rates increasing from 33% to 84% in the same period.^21^ Despite this national trend, 89.6% of patients in this cohort underwent ALND. Additionally, 90.2% underwent chemotherapy, whereas the national average in 2023 was 30%.^21^ Similarly, 88.3% of patients in this cohort received radiotherapy, with the national average being approximately 64%.^21^ These findings highlight the high-risk nature of this cohort.

Interestingly, despite this cohort’s intensive oncologic treatment profile, BCRL severity and progression varied considerably. ALND was significantly associated with higher ICG-NIFI stages, supporting existing evidence identifying ALND as a major risk factor for BCRL.^7,22,23^ Radiotherapy, an equally recognized risk factor for BCRL as well as (taxane-based) chemotherapy, were anticipated to be associated with BCRL severity and progression based on treatment intensity.^7,23–27^ However, no significant associations were found between treatment modalities, such as radiotherapy, chemotherapy (including taxane-based regimens), or hormone therapy, and BCRL severity or progression. These findings suggest that, although breast cancer treatment may trigger the onset of BCRL, its intensity may not directly influence the condition’s progression, indicating that individual predisposition or susceptibility may play a larger role.^26^ Identifying the extent of this individual predisposition is crucial as these treatments continue to be a key component of breast cancer treatment.

Moreover, lymphedema staging did not consistently align with the years since onset, suggesting that BCRL progression is variable and not solely dependent on the time since onset. Distinct patterns were observed in the distribution of affected body parts relative to lymphedema severity. Earlier stages more commonly involved the breast and flank, whereas higher stages showed pronounced involvement of the hand, lower arm, and upper arm, suggesting a trend in lymphatic dysfunction from proximal to distal regions.

Clinical progression of BCRL, defined by changes in symptoms over time, showed that lower ICG-NIFI stages were often associated with stable or gradually worsening symptoms, whereas higher stages exhibited more variable symptom patterns. Patients with stable lymphedema reported better functional outcomes and QoL, emphasizing the importance of symptom stability. Most patients were classified as moderate to severe on the Lymph-ICF questionnaires, emphasizing the significant impact of lymphedema on QoL and the need for targeted interventions for symptom stability.

Finally, notable differences were observed between the two staging systems being used to classify BCRL severity, ISL and ICG-NIFI. Although both aim to quantify BCRL severity, different aspects of lymphedema are being scored. ISL staging reports clinical signs of lymphedema, which can vary within a single limb, complicating interpretation and classification.^16^ In contrast, ICG-NIFI provides a real-time view of the lymphatic system, allowing for a more objective assessment of lymphatic dysfunction and subsequently classification of severity.^17^ These differences underscore the need for a unified classification system to ensure consistency in evaluating BCRL severity.

### Limitations and Suggestions for Future Research

This study’s findings are based on a highly selected patient population, limiting their generalizability. The study’s retrospective design and inherent data limitations necessitated the use of multivariate imputation by chained equations, whereas the small sample size also restricted subgroup analyses. Larger, prospective studies with more robust correction models are needed to validate these results and further explore variable interactions. Given the increasing influence of genetic makeup, future studies should consider expanding the scope of the data collected.

## Conclusions

This study offers a comprehensive profiling of patients with BCRL seeking microsurgical treatment, revealing patterns in its development not previously described. Patients presented with various stages of lymphedema, irrespective of time since onset or prior treatments, suggesting a variable progression timeline. Contrary to expectations, oncological treatment intensity did not appear to influence lymphedema severity or progression within this cohort. These findings challenge existing theories and suggest that BCRL severity and progression may depend more on biological predisposition than oncological treatment intensity. Although the highly selective nature of this patient population warrants cautious interpretation, these findings contribute to the growing understanding of BCRL and lay the groundwork for future studies to identify at-risk patients, allowing for implementation of targeted, individually tailored preventive strategies.

## Supplementary Information

Below is the link to the electronic supplementary material.

Supplementary file1 (DOCX 102 KB)

## Data Availability

The datasets generated and/or analyzed during the current study are not publicly available because of privacy or ethical restrictions but are available from the corresponding author on reasonable request.

## References

[1] Arnold M, Morgan E, Rumgay H, et al. Current and future burden of breast cancer: Global statistics for 2020 and 2040. *Breast*. 2022;66:15–23. 10.1016/j.breast.2022.08.010. 36084384 10.1016/j.breast.2022.08.010 PMC9465273

[2] DiSipio T, Rye S, Newman B, Hayes S. Incidence of unilateral arm lymphoedema after breast cancer: A systematic review and meta-analysis. *Lancet Oncol*. 2013;14(6):500–15. 10.1016/S1470-2045(13)70076-7. 23540561 10.1016/S1470-2045(13)70076-7

[3] Warren AG, Brorson H, Borud LJ, Slavin SA. Lymphedema: A comprehensive review. *Ann Plast Surg*. 2007;59(4):464–72. 10.1097/01.sap.0000257149.42922.7e. 17901744 10.1097/01.sap.0000257149.42922.7e

[4] Grada AA, Phillips TJ. Lymphedema: Pathophysiology and clinical manifestations. *J Am Acad Dermatol*. 2017;77(6):1009–20. 10.1016/j.jaad.2017.03.022. 29132848 10.1016/j.jaad.2017.03.022

[5] O’Donnell TFJ, Allison GM, Melikian R, Iafrati MD. A systematic review of the quality of clinical practice guidelines for lymphedema, as assessed using the appraisal of guidelines for research and evaluation II instrument. *J Vasc Surg Venous Lymphat Disord*. 2020;8(4):685–92. 10.1016/j.jvsv.2020.04.008. 32335331 10.1016/j.jvsv.2020.04.008

[6] Fu MR. Breast cancer-related lymphedema: Symptoms, diagnosis, risk reduction, and management. *World J Clin Oncol*. 2014;5(3):241–7. 10.5306/wjco.v5.i3.241. 25114841 10.5306/wjco.v5.i3.241 PMC4127597

[7] Gillespie TC, Sayegh HE, Brunelle CL, Daniell KM, Taghian AG. Breast cancer-related lymphedema: Risk factors, precautionary measures, and treatments. *Gland Surg*. 2018;7(4):379–403. 10.21037/gs.2017.11.04. 30175055 10.21037/gs.2017.11.04 PMC6107585

[8] Jørgensen MG, Toyserkani NM, Hansen FG, Bygum A, Sørensen JA. The impact of lymphedema on health-related quality of life up to 10 years after breast cancer treatment. *NPJ Breast Cancer*. 2021;7(1):70. 10.1038/s41523-021-00276-y. 34075045 10.1038/s41523-021-00276-y PMC8169644

[9] Fu MR, Ridner SH, Hu SH, Stewart BR, Cormier JN, Armer JM. Psychosocial impact of lymphedema: A systematic review of literature from 2004 to 2011. *Psychooncology*. 2013;22(7):1466–84. 10.1002/pon.3201. 23044512 10.1002/pon.3201 PMC4153404

[10] Sun Y, Shigaki CL, Armer JM. The influence of breast cancer related lymphedema on women’s return-to-work. *Womens Health (Lond)*. 2020;16:1745506520905720. 10.1177/1745506520905720. 32293984 10.1177/1745506520905720 PMC7160764

[11] Boccardo F, Fulcheri E, Villa G, et al. Lymphatic microsurgery to treat lymphedema: Techniques and indications for better results. *Ann Plast Surg*. 2013;71(2):191–5. 10.1097/SAP.0b013e31824f20d4. 23542829 10.1097/SAP.0b013e31824f20d4

[12] Campisi C, Davini D, Bellini C, et al. Lymphatic microsurgery for the treatment of lymphedema. *Microsurgery*. 2006;26(1):65–9. 10.1002/micr.20214. 16444753 10.1002/micr.20214

[13] Hill WKF, Deban M, Platt A, Rojas-Garcia P, Jost E, Temple-Oberle C. Immediate lymphatic reconstruction during axillary node dissection for breast cancer: A systematic review and meta-analysis. *Plast Reconstr Surg Glob Open*. 2022;10(5):e4291. 10.1097/GOX.0000000000004291. 35558135 10.1097/GOX.0000000000004291 PMC9084431

[14] Executive Committee of the International Society of Lymphology. The diagnosis and treatment of peripheral lymphedema: 2020 consensus document of the international society of lymphology. *Lymphology*. 2020;53(1):3–19. 32521126

[15] Jørgensen MG, Toyserkani NM, Hansen FCG, Thomsen JB, Sørensen JA. Prospective validation of indocyanine green lymphangiography staging of breast cancer-related lymphedema. *Cancers (Basel)*. 2021;13(7):1540. 10.3390/cancers13071540. 33810570 10.3390/cancers13071540 PMC8063087

[16] Devoogdt N, Van Kampen M, Geraerts I, Coremans T, Christiaens M. Lymphoedema functioning, disability and health questionnaire (lymph-ICF): Reliability and validity. *Phys Ther*. 2011;91(6):944–57. 10.2522/ptj.20100087. 21493748 10.2522/ptj.20100087

[17] Herdman M, Gudex C, Lloyd A, et al. Development and preliminary testing of the new five-level version of EQ-5D (EQ-5D-5L). *Qual Life Res*. 2011;20(10):1727–36. 10.1007/s11136-011-9903-x. 21479777 10.1007/s11136-011-9903-x PMC3220807

[18] Gummesson C, Atroshi I, Ekdahl C. The disabilities of the arm, shoulder and hand (DASH) outcome questionnaire: Longitudinal construct validity and measuring self-rated health change after surgery. *BMC Musculoskelet Disord*. 2003;4:11–11. 10.1186/1471-2474-4-11. 12809562 10.1186/1471-2474-4-11 PMC165599

[19] Chung KC, Pillsbury MS, Walters MR, Hayward RA. Reliability and validity testing of the michigan hand outcomes questionnaire. *J Hand Surg*. 1998;23(4):575–587. https://www.sciencedirect.com/science/article/pii/S0363502398800427. 10.1016/S0363-5023(98)80042-7. 10.1016/S0363-5023(98)80042-7 9708370

[20] Posit team PS, PBC. Rstudio: Integrated development environment for R. 2024.

[21] Netherlands Cancer Registry &, Netherlands Comprehensive Cancer Organisation (IKNL). NCR data & figures. https://iknl.nl/en/ncr/ncr-data-figures. Accessed 19-05-2025.

[22] DiSipio T, Rye S, Newman B, Hayes S. Incidence of unilateral arm lymphoedema after breast cancer: A systematic review and meta-analysis. *Lancet Oncol*. 2013;14(6):500–15. 10.1016/S1470-2045(13)70076-7. 23540561 10.1016/S1470-2045(13)70076-7

[23] Sayegh HE, Asdourian MS, Swaroop MN, et al. Diagnostic methods, risk factors, prevention, and management of breast cancer-related lymphedema: Past, present, and future directions. *Curr Breast Cancer Rep*. 2017;9(2):111–21. 10.1007/s12609-017-0237-8. 28894513 10.1007/s12609-017-0237-8 PMC5590641

[24] Swaroop MN, Ferguson CM, Horick NK, et al. Impact of adjuvant taxane-based chemotherapy on development of breast cancer-related lymphedema: Results from a large prospective cohort. *Breast Cancer Res Treat*. 2015;151(2):393–403. 10.1007/s10549-015-3408-1. 25940996 10.1007/s10549-015-3408-1 PMC4432026

[25] Zhang Z, Zhang X, Chen S, et al. Taxane-based chemotherapy and risk of breast cancer-related lymphedema: Protocol for a systematic review and meta-analysis. *Medicine (Baltimore)*. 2019;98(30):e16563. 10.1097/MD.0000000000016563. 31348280 10.1097/MD.0000000000016563 PMC6708704

[26] Naoum GE, Taghian AG. Regional lymph node radiation is not the main risk factor for breast cancer related lymphedema: Stop chasing radiation doses, fractionation or techniques-focus on axillary surgery de-escalation or prevention. *Int J Radiat Oncol Biol Phys*. 2023;117(2):461–4. 10.1016/j.ijrobp.2023.04.020. 37652608 10.1016/j.ijrobp.2023.04.020

[27] Abouegylah M, Elemary O, Munir A, Gouda MY, Arafat WO, Elzawawy S. Evaluation of the effect of axillary radiotherapy dose and the development of lymphedema in breast cancer patients. *Breast Care (Basel)*. 2022;17(4):364–70. 10.1159/000522243. 36156914 10.1159/000522243 PMC9453663

