# Supplementary material for: Breast Cancer-Related Lymphedema (BCRL): Comprehensive Characterization of Patients Seeking Microsurgical Treatment
Source: Ann Surg Oncol. 2025 Nov 18;33(3):2019–27. doi: 10.1245/s10434-025-18694-8 (PMC12901141; doi:10.1245/s10434-025-18694-8)
Supplement: Supplementary file 1 — Supplementary file1 (DOCX 102 KB) [file 10434_2025_18694_MOESM1_ESM.docx]

# Supplements

|  | **Overall** **(N=163)** |
| --- | --- |
| **Years since breast cancer diagnosis, median (IQR)** | 9.00 (IQR 7.0 - 13.0) |
| **Affected breast** |  |
| Right | 77 (47.2%) |
| Left | 81 (49.7%) |
| Bilateral | 5 (3.1%) |
| **T - stage** |  |
| Tis | 1 (0.6%) |
| T0 | 1 (0.6%) |
| T1 | 57 (35.0%) |
| T2 | 77 (47.2%) |
| T3 | 23 (14.1%) |
| T4 | 4 (2.5%) |
| **N - stage** |  |
| N0 | 28 (17.2%) |
| N1 | 67 (41.1%) |
| N2 | 46 (28.2%) |
| N3 | 21 (12.9%) |
| N4 | 1 (0.6%) |
| **M - stage** |  |
| M0 | 135 (82.8%) |
| M1 | 1 (0.6%) |
| Mx | 27 (16.6%) |
| **Primary tumor location, n (%)** |  |
| Upper outer quadrant | 107 (65.6%) |
| Upper inner quadrant | 24 (14.7%) |
| Lower inner quadrant | 9 (5.5%) |
| Centrally behind nipple | 5 (3.1%) |
| Multifocal | 17 (10.4%) |
| Unknown | 1 (0.6%) |
| **Primary tumor diameter (mm), median (IQR)** | 23.0 (IQR 15.0 - 35.0) |
| **ER pos (≥10%), n (%)** | 110 (67.5%) |
| **Her2Neu pos (IHC 3+ or amplification), n (%)** | 28 (17.2%) |
| **Germline DNA mutations, n (%)** |  |
| *BRCA1* | 8 (4.9%) |
| *BRCA2* | 2 (1.2%) |
| *CHEK21100delC* | 1 (0.6%) |
| No DNA mutations | 152 (93.3%) |
| **Breast surgery (yes), n (%)** | 162 (99.4%) |
| **Type of breast surgery** |  |
| Mastectomy | 97 (59.9%) |
| Lumpectomy | 65 (40.1%) |
| **ALND (total), n (%)** | 146 (89.6%) |
| **SLNB (total), n (%)** | 58 (35.6%) |
| **Lymph nodes removed, median (IQR)** | 14.0 (IQR 9.0 - 20.5) |
| **Reconstructive surgery** |  |
| Primary reconstructive surgery | 21 (12.9%) |
| Secondary reconstructive surgery | 60 (36.8%) |
| No reconstructive surgery | 82 (50.3%) |
| **Chemotherapy (yes), n (%)** | 147 (90.2%) |
| **Adjuvant/Neoadjuvant** |  |
| Adjuvant | 95 (64.6%) |
| Neoadjuvant | 51 (34.7%) |
| Neoadjuvant and adjuvant | 1 (0.7%) |
| **Chemotherapy sessions, median (IQR)** | 6.0 (IQR 5.5 - 8.0) |
| **Type of chemotherapy, n (%)** |  |
| Anthracycline and taxane based schedules | 103 (70.1%) |
| Solely anthracycline based agents | 39 (26.5%) |
| Solely taxane based agents | 5 (3.4%) |
| **Taxane-based chemotherapy (yes), n (%)** | 108 (66.3%) |
| **Platinum-based chemotherapy (yes), n (%)** | 8 (4.9%) |
| **Antihormonal therapy (yes), n (%)** | 110 (67.5%) |
| **Anti-HER2 targeted therapy (yes), n (%)** | 19 (11.6%) |
| **PARP inhibitor targeted therapy (yes), n (%)** | 3 (13.1%) |
| **CDK4/6 inhibitor therapy (yes), n (%)** | 1 (4.3%) |
| **Radiotherapy (yes), n (%)** | 143 (87.7%) |
| **Irradiation site / Targeted regions** |  |
| Breast | 29 (20.3%) |
| Breast and axillary lymph nodes | 30 (20.9%) |
| Chest wall | 31 (21.7%) |
| Chest wall and axillary lymph nodes | 52 (36.4%) |
| Periclavicular lymph nodes and axillary lymph nodes | 1 (0.7%) |
| **Regional nodal irradiation (RNI), (yes) n (%)** | 83 (58%) |
| **Radiation therapy boost (yes), n (%)** | 45 (31.5%) |
| **Total radiation dosage (Gy), median (IQR)** | 50.0 Gy (IQR 40.0-55.9) |
| **Number of radiotherapy sessions, median (IQR)** | 25.0 (IQR 15.0-25.0) |

**Supplementary Table 1: Oncological characteristics and treatment history of patients with breast cancer-related lymphedema (BCRL).**


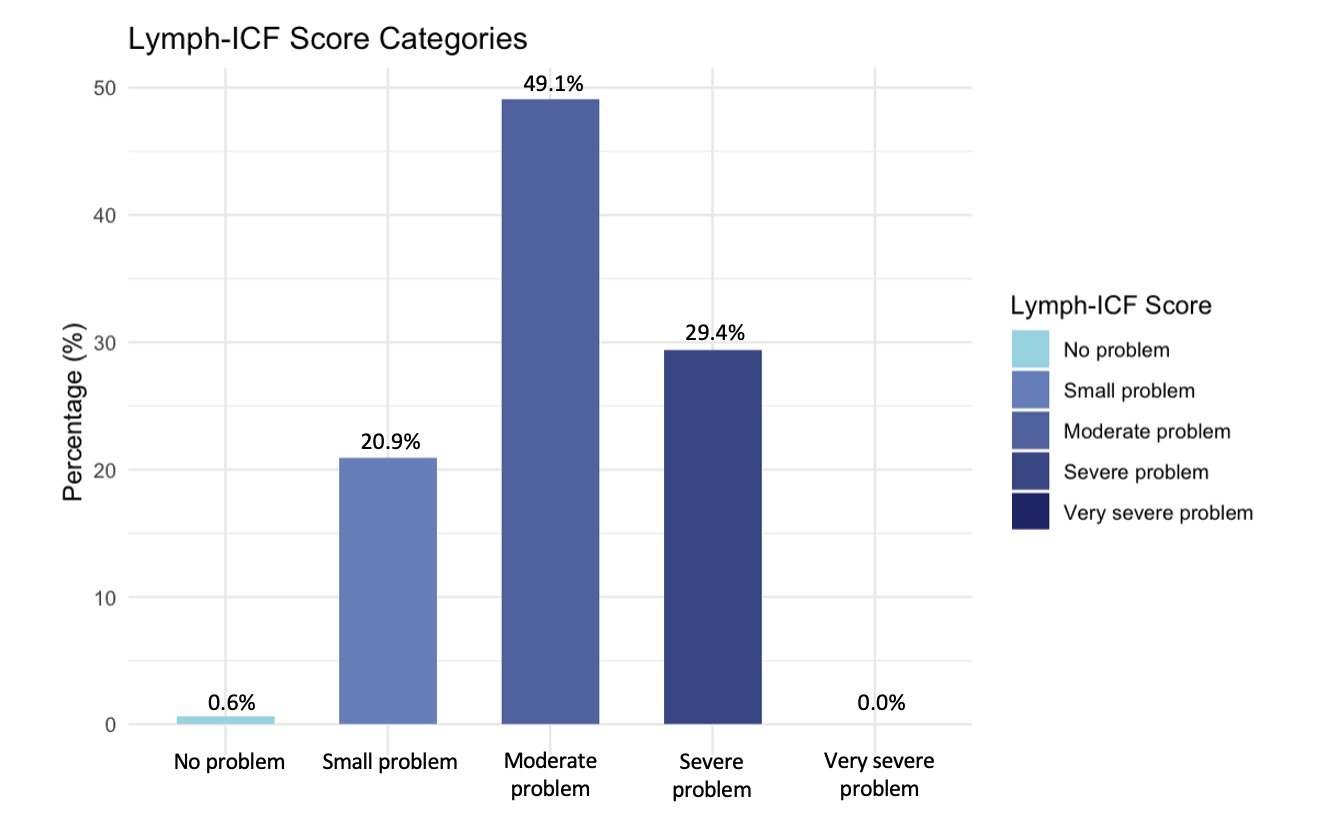


Supplementary Figure 1: Lymph-ICF categories based on total score.
